# Metabolic syndrome associated with higher glycemic variability in type 1 diabetes: A multicenter cross-sectional study in china

**DOI:** 10.3389/fendo.2022.972785

**Published:** 2022-09-20

**Authors:** Keyu Guo, Liyin Zhang, Jianan Ye, Xiaohong Niu, Hongwei Jiang, Shenglian Gan, Jian Zhou, Lin Yang, Zhiguang Zhou

**Affiliations:** ^1^ National Clinical Research Center for Metabolic Diseases, Key Laboratory of Diabetes Immunology, Ministry of Education, and Department of Metabolism and Endocrinology, The Second Xiangya Hospital of Central South University, Changsha, China; ^2^ Department of Endocrinology, Heji Hospital Affiliated to Changzhi Medical College, Changzhi, China; ^3^ Department of Endocrinology, The First Affiliated Hospital and College of Clinical Medicine of Henan University of Science and Technology, Luoyang, China; ^4^ Department of Endocrinology, The First People’s Hospital of Changde City, Changde, Hunan, China; ^5^ Department of Endocrinology and Metabolism, Shanghai Jiao Tong University Affiliated Sixth People’s Hospital, Shanghai, China

**Keywords:** type 1 diabetes mellitus, metabolic syndrome, continuous glucose monitoring, glycemic control, glycemic profile

## Abstract

**Aims:**

The comorbidity of metabolic syndrome (MetS) and type 1 diabetes mellitus (T1DM) is an obstacle to glucose control in patients with T1DM. We compared glycemic profiles using continuous glucose monitoring (CGM) systems in patients with T1DM with or without MetS.

**Methods:**

This was a multicenter cross-sectional study of patients with T1DM (N = 207) with or without MetS. CGM data were collected from study enrollment until discharge during a 1-week study session. We analyzed baseline HbA1c, average glucose, estimated HbA1c, time in range (TIR), time above range (TAR), time below range (TBR), coefficient of variation (CV), postprandial glucose excursions (PPGE) and other glycemic variability (GV) metrics. Logistic regression was developed to investigate the association between MetS and CGM metrics.

**Results:**

The results showed higher average baseline HbA1c levels, and a higher percentage of patients with baseline HbA1c levels ≥7.5%, in the T1DM with MetS group. Furthermore, MetS was associated with GV, which indicated a higher CV in patients with T1DM with MetS. However, our results showed that TAR, TIR, TBR and other GV metrics were comparable between the two groups. The T1DM with MetS group also had a higher proportion of patients with high CV (≥ 36%) than the group without MetS. In multivariable logistic regression analysis, the presence of MetS was a risk factor for high CV (≥ 36%) in our study participants.

**Conclusions:**

T1DM patients with MetS in our study had better β-cell function. However, MetS was associated with worse glycemic control characterized by higher GV and HbA1c levels. Efforts should be expanded to improve treatment of MetS in patients with T1DM to achieve better glycemic control.

## Introduction

Type 1 diabetes mellitus (T1DM) is a progressive disease that results from loss of islet β-cell function, which leads to high glucose variability. Metabolic syndrome (MetS) is characterized by a cluster of metabolic disorders, including glucose intolerance, central obesity, hypertension, and dyslipidemia, and insulin resistance plays a key role in the in the progression of the MetS. The global incidence of MetS in patients with T1DM has increased with the increased incidence of T1DM worldwide (1–4). Comorbid MetS and T1DM, possesses the features of both insulin resistance and insulin deficiency, presents a significant challenge to efficient glucose control in patients with T1DM. Studies have shown that obesity or MetS in diabetes results in an increased risk of cardiovascular death in patients with diabetes, and is a leading cause of diabetes-related disability adjusted life years (5–8). Several studies have evaluated the impact of MetS on development of chronic complications in patients with T1DM, and showed that MetS with T1DM is a risk factor for macrovascular and microvascular complications (9–13). Patients with T1DM and MetS are at higher risk for diabetic complications compared with T1DM patients without MetS, independent of HbA1c values (4, 14). Moreover, the rate of complications in patients with well-controlled T1DM with MetS was higher than that in patients with T1DM without MetS, regardless of HbA1c levels (1, 15). Thus, HbA1c alone does not explain the increased rate of complications in patients with T1DM and MetS. HbA1c is the gold standard for evaluating long‐term glycemic control. However, a wide range of mean glucose concentrations and glucose profiles can be associated with a given HbA1c level, making this measure potentially misleading (16). Therefore, further research is required regarding the relationship between MetS and dynamic glycemic profiles in T1DM. The use of continuous glucose monitoring (CGM) systems has enabled the collection of a number of metrics to allow for comprehensive assessment of glycemic profiles in patients with T1DM (17). Continuous recording of glucose values can be used as a reference to determine dosing regimens to adjust glucose-lowering treatment. Therefore, our study aimed to compare dynamic glycemic profiles using CGMs in patients with T1DM with or without MetS to provide insights into the strategy for better diabetes management.

## Methods

### Study population

This was a retrospective, multicenter, cross-sectional study of patients with T1DM who used a CGM system (Medtronic, Northridge, CA) from October 2019 to December 2021. Patients were recruited from 5 tertiary care hospitals in 4 provincial administration areas in China: 1) The Second Xiangya Hospital of Central South University, Hunan; 2) Shanghai Jiao Tong University Affiliated Sixth People’s Hospital, Shanghai; 3) Heji Hospital Affiliated to Changzhi Medical College, Shanxi; 4) The First Affiliated Hospital and College of Clinical Medicine of Henan University of Science and Technology, Henan; and 5) The First People’s Hospital of Changde City, Hunan. The study protocol was approved by the Ethics Committees of the five hospitals mentioned above, in accordance with the principles of the Helsinki Declaration. Written informed consent was obtained from each participant.

### Inclusion and exclusion criteria

Patients with T1DM were included based on the following criteria: 1) met the 1999 WHO diagnostic criteria for diabetes; 2) insulin-dependent treatment from diagnosis; and 3) treated with multiple daily insulin injections (MDI) or continuous subcutaneous insulin infusion (CSII). The exclusion criteria was as follows: 1) use of other types of CGMsystems; 2) recent complications, including ketoacidosis, acute infection, chronic infection, surgery, trauma and other stress states; 3) long-term use of glucocorticoids or immunomodulators; 4) unwilling to wear CGM equipment or allergic to equipment; 5) acute and chronic hepatic and renal insufficiency; and 6) presence of other autoimmune diseases, such as abnormal thyroid function.

Diagnosis of MetS was based on the updated NCEP-ATPIII criteria (18). All subjects were assumed to have hyperglycemia. Participants were diagnosed with MetS if they met two or more of the following criteria (1): central obesity (waist circumference ≥ 90 cm in men or ≥ 80 cm in women); (2) hypertension (systolic blood pressure ≥ 130 and/or diastolic blood pressure ≥ 85 mmHg and/or a history of antihypertensive therapy); (3) hypertriglyceridemia (serum triglyceride levels ≥ 1.7 mmol/L); and (4) low HDL-c level (HDL-c < 1.0 mmol/L in men or < 1.3 mmol/L in women).

### Data collection

CGM was performed using iPro2^®^ as the recorder and an Enlite^®^ glucose sensor (Medtronic, Northridge, CA). The CGM system was inserted in the arm or abdominal area according to the manufacturer’s guidelines. Standard POC capillary blood glucose measurements were performed using Gold AQ glucometers (Sinocare, China) three times daily before breakfast, lunch, and dinner to calibrate the CGMs. CGM data were collected from study enrollment until discharge during a week-long study session, and all participants were instructed to adhere to a standard diet. The patients were instructed to keep a regular diet, avoid strenuous exercise, and maintain detailed records of diet, exercise, and insulin dose. In the present study, we analyzed average glucose, estimated HbA1c (eHAb1c), glucose variability (calculated as the coefficient of variation (CV); mean amplitude of glycemic excursions (MAGE); mean of daily differences (MODD); continuous glucose overlapping net glycemic action (CONGA); low blood glucose index (LBGI); high blood glucose index (HBGI); and postprandial glucose excursions (PPGE)), time in range (TIR, 3.9-10.0 mmol/l), time above range (TAR, > 10.0 mmol/l), and time below range (TBR, < 3.9 mmol/l).

### Propensity score matching

All participants were then categorized into two groups based on the presence of MetS. Of the 604 patients with T1DM, 72 participants were diagnosed with MetS. To reduce the impact of age, sex, and duration of diabetes on glycemic variability, 532 participants without MetS were matched in a 2:1 ratio with 72 participants with MetS using propensity score matching. First, 532 patients without MetS were extracted. Nearest neighbor matching was used as the matching algorithm. The threshold was set at the 0.0015 level. A propensity score was generated using a logistic regression model. The covariates used for matching were age, sex, and duration of diabetes. Finally, 135 patients without MetS were matched in a 2:1 ratio with 72 patients with MetS based on propensity scores. There were no differences in age, sex, or duration of diabetes between the two groups (all *P* > 0.05). Therefore, 207 participants with T1DM were included in the final analysis.

### Statistical analysis

Normally distributed measurement data were presented as the mean ± standard deviation, and skewed data after normality testing (Shapiro-Wilk test) are presented as the median and interquartile range (IQR). Independent samples t-test or the Mann–Whitney U test was used to compare differences between groups. Spearman rank correlation was used for correlation analysis. Assessment of differences in proportions between two groups was done by using a chi-square test. Multiple logistic regression was performed to assess the odds ratio (OR) and 95% confidence interval (CI) for the associations between the presence of MetS and glycemic variability after adjusting for confounding variables such as age, BMI, duration of diabetes, daily insulin dosage, HbA1c, FCP, 2hCP, and other known risk factors for glycemic variability. All tests were two-tailed, and P < 0.05 was considered statistically significant. SPSS 26.0 software (IBM Corporation, Armonk, NY, USA) was used for statistical analysis.

## Results

### Characteristics of the study patients

Of the 207 participants enrolled in the analysis, 70.0% were female. The mean age was 43.0 (27.5, 58.0) years. The average duration of diabetes was 6.0 (1.5, 12.0) years. The mean BMI level was 21.9 ± 3.3 kg/m^2^. The patients were divided into the following two groups based on the presence of MetS: the MetS group (n = 72) and the non-MetS group (n = 135). As shown in **Table 1**, age, sex, diabetes duration, and insulin schema were similar between the two groups (all *P* > 0.05). However, patients in the MetS group had higher BMI (23.5 vs 21.1 kg/m^2^, *P* < 0.001) and WHR (0.89 vs 0.83, *P* < 0.001) values than those in the non-MetS group. Moreover, the SBP and DBP in the MetS group were significantly higher than those in the non-MetS group (both *P* < 0.001). Furthermore, bolus insulin dose was higher (0.40 vs 0.34 U/kgd, *P* = 0.035), FCP was higher in patients with MetS (48.6 vs 25.2 pmol/L, *P* = 0.017), and HbA1c was much higher in the MetS group (9.1% vs 7.9%, *P* = 0.004). In addition, TG was higher and HDL-c was lower in the MetS group (both *P* < 0.001).

**Table 1:** Characteristics of all participants, and patients with or without MetS.

	All patients (*n* = 207)	Non-MetS group (*n* = 135)	MetS group (*n* = 72)	*P value*
Sex (M/F)	62/145	39/96	23/49	0.648
Age (years)	43.0 (27.5, 58.0)	40.0 (27.5, 56.0)	44.5 (27.5, 63.5)	0.200
Diabetes duration (years)	6.0 (1.5, 12.0)	5.4 (1.3, 11.6)	7.0 (1.8, 12.8)	0.761
BMI (kg/m^2^)	21.9 ± 3.3	21.1 ± 2.9	23.5 ± 3.5	<0.001
WHR	0.85 ± 0.07	0.83 ± 0.06	0.89 ± 0.06	<0.001
Insulin schema (MDI/CSII)	184/23	116/19	68/4	0.251
Insulin dose (U/kg**·**d)	0.56 (0.42, 0.71)	0.55 (0.44, 0.69)	0.62 (0.39, 0.75)	0.162
Basal insulin dose (U/kg·d)	0.19 (0.11, 0.26)	0.19 (0.11, 0.25)	0.19 (0.13, 0.27)	0.519
Bolus insulin dose (U/kg·d)	0.35 (0.26, 0.51)	0.34 (0.25, 0.49)	0.40 (0.27, 0.56)	0.035
SBP (mmHg)	121 ± 19	116 ± 14	131 ± 22	<0.001
DBP (mmHg)	76 ± 10	74 ± 9	81 ± 11	<0.001
HbA1c (%)	8.3 (7.2, 10.1)	7.9 (7.0, 9.5)	9.1 (7.6, 10.9)	0.004
HbA1c ≤ 7.5% (%)	35.7	40.9	25.4	0.031
FCP (pmol/L)	34.6 (16.5, 107.2)	25.2 (16.5, 85.9)	48.6 (16.5, 130.1)	0.017
2hCP (pmol/L)	89.0 (16.9, 288.6)	73.4 (16.5, 311.1)	131.2 (25.1, 272.7)	0.406
FBG (mmol/L)	8.5 (6.0, 11.8)	7.8 (5.8, 11.2)	8.9 (6.6, 12.9)	0.065
2hBG (mmol/L)	13.5 (10.7, 17.4)	13.3 (10.4, 17.1)	14.1 (11.6, 18.6)	0.178
TC (mmol/L)	4.6 ± 1.1	4.6 ± 1.0	4.7 ± 1.2	0.454
TG (mmol/L)	0.8 (0.6, 1.3)	0.7 (0.6, 1.0)	1.2 (0.8, 2.1)	<0.001
HDL-c (mmol/L)	1.5 ± 0.5	1.6 ± 0.4	1.2 ± 0.4	<0.001
LDL-c (mmol/L)	2.7 ± 0.9	2.6 ± 0.8	2.9 ± 1.1	0.067

Data are shown as the mean ± SD, median (IQR), or frequency.

MetS, metabolic syndrome; BMI, body mass index; WHR, waist to hip ratio; MDI, multiple daily insulin injection; CSII, continuous subcutaneous insulin infusion; SBP, systolic pressure; DBP, diastolic pressure; HbA1c, hemoglobin A1c; FCP, fasting C-peptide, 2hCP, 2-hour postprandial C-peptide; FBG, fasting blood glucose, 2hBG, 2-hour postprandial blood glucose; TC, total cholesterol; TG, triglyceride; HDL-c, high-density lipoprotein cholesterol; LDL-c, low-density lipoprotein cholesterol.

P values for the MetS group vs. the non-MetS group.

### CGM-derived metrics of the study patients

CGM-derived metrics showed that TAR, TIR, and TBR were comparable between the two groups (**Table 2**). However, glycemic variability-related parameters such as SD (3.4 vs 3.0 mmol/L, *P* = 0.03) and CV (36.5% vs 32.1%, *P* = 0.01) of glucose levels and higher proportion of patients with high CV (≥ 36%) (51.4% vs 28.0%, *P* = 0.001) were significantly higher in patients with MetS. The proportion of TIR ≥ 70% between the two groups was compared, and the proportion was lower in the MetS group, but the difference was not statistically significant (24.3% vs 37.1%, *P* = 0.064).

**Table 2:** CGM-derived metrics for all participants, and patients with or without MetS .

	All patients (*n* = 207)	Non-MetS group (*n* = 135)	MetS group (*n* = 72)	*P value*
eHbA1c (%)	7.6 (6.7, 8.4)	7.6 (6.6, 8.5)	7.7 (6.8, 8.2)	0.565
%TAR (>10.0mmol/L)	39.4 (20.8, 55.7)	38.9 (15.4, 58.6)	39.4 (25.5, 51.5)	0.642
TAR < 25% (%)	32.7	37.1	24.3	0.064
%TIR (3.9-10mmol/L)	58.1 (41.4, 75.9)	60.1 (40.3, 79.6)	57.7 (47.2, 69.3)	0.581
TIR ≥ 70% (%)	32.7	37.1	24.3	0.064
%TBR (<3.9mmol/L)	1.1 (0, 3.9)	1.0 (0, 4.0)	1.5 (0, 3.6)	0.695
TBR < 4% (%)	76.7	75.6	78.6	0.652
Mean glucose (mmol/L)	9.5 (7.9, 10.9)	9.4 (7.7, 11.0)	9.7 (8.2, 10.5)	0.531
SD (mmol/L)	3.1 (2.4, 3.9)	3.0 (2.4, 3.7)	3.4 (2.5, 4.3)	0.030
MAGE (mmol/L)	6.6 (5.1, 8.4)	6.4 (5.0, 7.9)	7.0 (5.2, 8.8)	0.149
CV (%)	33.3 (27.4, 38.4)	32.1 (27.3, 36.6)	36.5 (27.6, 41.9)	0.010
CV < 36% (%)	63.9	72.0	48.6	0.001
CV ≥ 36% (%)	36.1	28.0	51.4	0.001
LBGI	2.4 (1.2, 4.1)	2.3 (1.1, 4.1)	2.6 (1.5, 4.4)	0.217
HBGI	10.1 (6.1, 15.5)	9.5 (5.2, 15.6)	10.9 (7.1, 14.5)	0.219

Data are shown as the median (IQR) or frequency.

MetS, metabolic syndrome; CGM, continuous glucose monitoring; eHbA1c, estimated HbA1c; TAR, time above range; TIR, time in range; TBR, time below range; SD, standard deviation of glucose; MAGE, mean amplitude of glucose excursions; CV, coefficient of variation; LBGI, low blood glucose index; HBGI, high blood glucose index.

P values for the MetS group vs. the non-MetS group.

**Figure 1:**
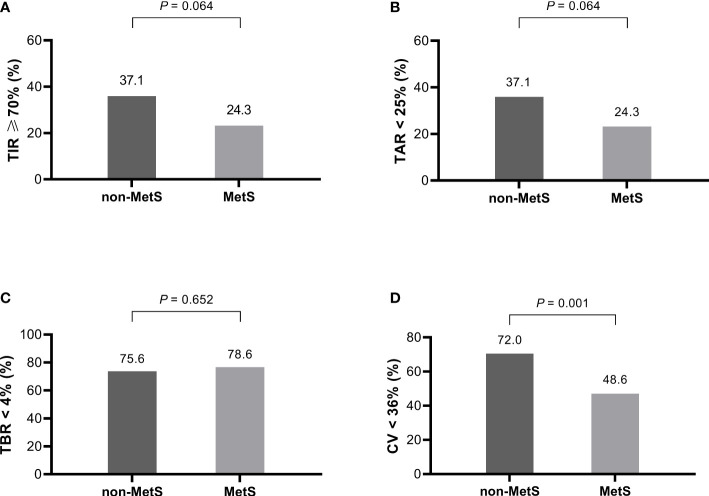
The proportion of patients with and without MetS who achieved the CGM targets (*n* = 207). **(A)** TIR ≥ 70%, **(B)** TAR < 25%, **(C)** TBR < 4%, **(D)** CV <36%. MetS, metabolic syndrome; TIR, time in range; TAR, time above range; TBR, time below range; CV, coefficient of variation.

The rates of achieving the CGM targets was further analyzed shown in **Figure 1**. The targets were set according to the international consensus guidelines on CGM use. The MetS group had significantly lower proportion of CV< 36%, and lower proportions, though not statistically significant, of TIR ≥ 70% and TAR < 25%. In contrast, proportion of TBR < 4% was comparable between the two groups. There were no differences in PPGE for breakfast (PPGE-B), lunch (PPGE-L), or dinner (PPGE-D) between the two groups.

### Associations between the presence of MetS and glycemic variability

To investigate the relationship between the presence of MetS and glycemic variability, multivariate logistic regression analysis was performed. As shown in **Table 3**, the odds ratio (OR) for the presence of MetS was 2.669 (95% CI: 1.443–4.936) for CV ≥ 36% in the model adjusted for age, sex, and duration of diabetes (Model 1). The association remained significant after further adjustment for BMI and daily insulin dosage (Model 2) (OR: 2.152, 95% CI: 1.089–4.253). Moreover, after further adjusting for common clinical parameters that affect glycemic variability such as HbA1c, FCP, and 2hCP, the model remained significant, which indicated that the presence of MetS was a risk factor for glycemic variability in our study participants.

**Table 3:** Association between the presence of MetS and glycemic variability.

	OR (95% CI)	*P* value
Model 1	2.669 (1.443, 4.936)	0.002
Model 2	2.152 (1.089, 4.253)	0.027
Model 3	2.418 (1.173, 4.987)	0.017
Model 4	3.902 (1.692, 8.995)	0.001
Model 5	3.019 (1.164, 7.829)	0.023

Model 1: adjusted for age, sex, and duration of diabetes.

Model 2: adjusted for model 1 plus BMI and daily insulin dosage.

Model 3: adjusted for model 2 plus HbA1c.

Model 4: adjusted for model 3 plus FCP.

Model 5: adjusted for model 4 plus 2hCP.

OR, odds ratio; CI, confidence interval.

## Discussion

This was the first study to investigate the relationship between MetS status and dynamic glycemic profile in patients with T1DM using CGMs. Although the patients with T1DM with MetS in our study had better β-cell function, MetS was associated with worse glycemic control characterized by higher GV and HbA1c level. In addition, MetS was inversely associated with TIR, and positively associated with TAR, although these results were not statistically significant.

The results showed higher average baseline HbA1c levels and higher percentages of patients that presented with baseline HbA1c levels ≥7.5% in the T1DM with MetS group than in the T1DM without MetS group. CGM data were collected from study enrollment until discharge during a week-long study session. There were no differences in eHbA1c or mean blood glucose between the two groups during the study. Furthermore, eHbA1c was lower than baseline HbA1c in both groups. The patients were instructed to follow a regular diet, avoid strenuous exercise, and maintain detailed records of diet, exercise, and insulin dosing. These instructions objectively resulted in lifestyle improvement, which may have improved mean blood glucose levels. Therefore, we hypothesized that MetS may play a critical role in GV in patients with T1DM. New therapeutic strategies are required to treat patients with T1DM and MetS to allow for better glycemic control.

We found that MetS was associated with GV, suggesting higher average SD and CV values in patients with T1DM patients with MetS. Patients with T1DM patients with MetS also had a higher prevalence of high CV (>36%) than patients without MetS. However, other measures of GV were not statistically significant between the groups. There are two types of GV, long-term GV is usually assessed using long-term HbA1c. Short-term GV is based on the intraday and interday variability in blood glucose. In this study, we evaluated short-term GV. Studies have shown that short-term and long-term GV are associated with onset and progression of chronic diabetic complications in T2DM (19–23). However, few studies have shown that GV was associated with diabetic complications in T1DM, and the results are inconsistent (20, 22, 24–27). Insulin resistance and the underlying pathophysiological mechanisms of insulin resistance are major contributors to development of MetS. Development of insulin resistance in patients with T1DM has led to the emergence of a distinct phenotype of mixed T1DM and T2DM (28). Therefore, it is likely that high GV is associated with development and progression of diabetic vascular complications in patients with T1DM with MetS. Patients with T1DM and MetS had a higher rate of complications than those without MetS who had identical HbA1c values (4, 14). Moreover, the rate of complications in patients with well-controlled T1DM with MetS was higher than that in patients with T1DM without MetS, regardless of glycemic control (1, 15). Furthermore, studies have shown that GV influences endothelial function in individuals with MetS, independent of diabetes, which may explain increased complications in patients with T1DM and MetS (29–31). HbA1c alone does not explain the increased rate of complications in patients with T1DM and MetS, and GV may play a role in increased complications in patients with T1DM and MetS. However, there is no direct evidence so far that indicates the relationship between increase GV in T1DM patients and diabetic complications in this paper. Therefore, long-term prospective studies are needed to characterize the role of GV in onset and progression of diabetic complications in T1DM patients with MetS. Postprandial hyperglycemia is a main cause of GV, which is closely related to risk of complications in patients with diabetes. However, our study did not show an association between PPGE and higher GV in patients with T1DM with MetS. The mechanism by which MetS affects GV in patients with T1DM requires further study.

The patients with T1DM and MetS in our study had better β-cell function. However, blood glucose control and insulin dosing in patients without MetS were superior to those in the MetS group. But we know now that individuals with better β-cell function had less GV than patients with lower residual C-peptide, our previous research also verified this conclusion as well (17). Studies have shown that insulin resistance plays a key role in the in the progression of the MetS, so T1DM patients with MetS sustained losses of insulin and insulin resistance in the meantime. Insulin resistance is a state in which higher than normal concentration of insulin is needed for individuals, leading directly to hyperinsulinemia, as daily insulin dose in patients with MetS were higher to those in the without MetS group in our study. Insulin resistance inhibited lipolysis in adipose tissu*e*, impaired glucose uptake by muscle and inhibited gluconeogenesis in liver, which causes ineffective blood glucose control in such patients (32). Recent works have already revealed a significant association between GV and insulin resistance (33–36). In our study the negative effects of insulin resistance in patient with MetS seem to exceed the corresponding positive effects of a narrow majority of fasting C-peptide. To investigate the relationship between the presence of MetS and glycemic variability, further multivariate logistic regression adjusting for common clinical parameters that affect glycemic variability (BMI, daily insulin dosage, HbA1c, FCP, and 2hFCP) indicated that the presence of MetS was a risk factor for glycemic variability in our study participants.

T1DM is a heterogeneous disease, and patients with T1DM with MetS account for a considerable proportion of the total population of individuals with T1DM. Development of precision medicine has allowed for better management of T1DM in patients with comorbid MetS. Our results indicated that insulin therapy alone cannot effectively achieve glycemic control in patients with MetS. At present insulin resistance is still considered as the core of development of MetS. In order to improve insulin resistance, it is essential for T1DM patients with MetS to promote a healthy lifestyle through a balanced diet, appropriate health habits and physical activity. Moreover, the common oral antidiabetic agents such as metformin, rosiglitazone, SGLT-2i (sodium dependent glucose transporters 2 inhibitor) and GLP-1RA (glucagon like peptide 1 receptor activation agents) have been widely used to improve insulin resistance in T2DM patients. To our satisfaction, studies display, from the present, metformin, rosiglitazone, SGLT2i or GLP-1RA also can improve insulin sensitivity in patients with T1DM (37–43). However, anti-hyperglycemic effect of those combination therapy awaits further study and profit always comes with risks (44–46). In order to get profits and avoid risks maximally, more precise interpretation of glucose dynamics could facilitate development of improved therapeutic strategies to manage T1DM in individuals with MetS.

Our study was subject to the following limitations. Although our study was a national multicenter study, it was cross-sectional, had a small sample size, and included five high-ranking hospitals. In addition, use of CGM systems required informed consent, which may have led to selection bias. Moreover, our paper is the lack of the analysis of physical activity, sleep quality, dietary habits, change in diet and insulin dose and other potential factors might affect the GV. Finally, our study only evaluated short-term GV and CGM was conducted for only a week. However, the optimal sampling duration for CGM to determine long-term glycemic control is recommended to be 14 days. Therefore, a cohort study with a larger sample size is needed to explore short-term and long-term GV in patients with T1DM with MetS, and to characterize the relationship between GV and clinical outcomes in patients with T1DM and MetS.

## Conclusion

This was the first study to show that MetS was associated with worse glycemic control using CGMs in T1DM patients. Although the patients with T1DM with MetS in our study had better β-cell function and higher bolus insulin dose, MetS was associated with worse glycemic control characterized by higher glycemic variability and HbA1c level. Efforts should be expanded to improve treatment of MetS in patients with T1DM to achieve better glycemic control.

## Data availability statement

The raw data supporting the conclusions of this article will be made available by the authors, without undue reservation.

## Ethics statement

The studies involving human participants were reviewed and approved by The Second Xiangya Hospital of Central South University, Hunan; Shanghai Jiao Tong University Affiliated Sixth People’s Hospital, Shanghai; Heji Hospital Affiliated to Changzhi Medical College, Shanxi; The First Affiliated Hospital and the College of Clinical Medicine of Henan University of Science and Technology, Henan and The First People’s Hospital of Changde City, Hunan. The patients/participants provided their written informed consent to participate in this study

## Author contributions

LY and ZZ designed the study. KG and LZ conducted the data analyses, and drafted the initial manuscript. KG, LZ, JY, XN, HJ, SG and JZ collected data. All authors reviewed and revised the manuscript, approved the final manuscript as submitted, and agreed to be accountable for all aspects of the work.

## Funding

This work was supported by the National Key R&D Program of China (Grant No. 2018YFC2001005) to LY.

## Conflict of interest

The authors declare that this research was conducted in the absence of any commercial or financial relationships that could be construed as a potential conflict of interest.

## Publisher’s note

All claims expressed in this article are solely those of the authors and do not necessarily represent those of their affiliated organizations, or those of the publisher, the editors and the reviewers. Any product that may be evaluated in this article, or claim that may be made by its manufacturer, is not guaranteed or endorsed by the publisher.
